# HIV and SARS-CoV-2 infection in postpartum Kenyan women and their infants

**DOI:** 10.1371/journal.pone.0278675

**Published:** 2023-01-17

**Authors:** Emily R. Begnel, Bhavna H. Chohan, Ednah Ojee, Judith Adhiambo, Prestone Owiti, Vincent Ogweno, LaRinda A. Holland, Carolyn S. Fish, Barbra A. Richardson, Adam K. Khan, Rabia Maqsood, Efrem S. Lim, Manish Sadarangani, Dara A. Lehman, Jennifer Slyker, John Kinuthia, Dalton Wamalwa, Soren Gantt

**Affiliations:** 1 Department of Global Health, University of Washington, Seattle, Washington, United States of America; 2 Kenya Medical Research Institute, Nairobi, Kenya; 3 Department of Paediatrics and Child Health, University of Nairobi, Nairobi, Kenya; 4 Center for Fundamental and Applied Microbiomics, Biodesign Institute, Arizona State University, Tempe, Arizona, United States of America; 5 Division of Human Biology, Fred Hutchinson Cancer Center, Seattle, Washington, United States of America; 6 Department of Biostatistics, University of Washington, Seattle, Washington, United States of America; 7 School of Life Sciences, Arizona State University, Tempe, Arizona, United States of America; 8 Vaccine Evaluation Center, BC Children’s Hospital Research Institute, Vancouver, British Columbia, Canada; 9 Department of Pediatrics, University of British Columbia, Vancouver, British Columbia, Canada; 10 Department of Epidemiology, University of Washington, Seattle, Washington, United States of America; 11 Department of Research and Programs, Kenyatta National Hospital, Nairobi, Kenya; 12 Département de Microbiologie, Infectiologie et Immunologie, Université de Montréal, Centre de Recherche du CHU St-Justine, Montréal, Québec, Canada; Washington State University, UNITED STATES

## Abstract

**Background:**

HIV may increase SARS-CoV-2 infection risk and COVID-19 severity generally, but data are limited about its impact on postpartum women and their infants. As such, we characterized SARS-CoV-2 infection among mother-infant pairs in Nairobi, Kenya.

**Methods:**

We conducted a nested study of 62 HIV-uninfected and 64 healthy women living with HIV, as well as their HIV-exposed uninfected (N = 61) and HIV-unexposed (N = 64) infants, participating in a prospective cohort. SARS-CoV-2 serology was performed on plasma collected between May 1, 2020-February 1, 2022 to determine the incidence, risk factors, and symptoms of infection. SARS-CoV-2 RNA PCR and sequencing was also performed on available stool samples from seropositive participants.

**Results:**

SARS-CoV-2 seropositivity was found in 66% of the 126 mothers and in 44% of the 125 infants. There was no significant association between SARS-CoV-2 infection and maternal HIV (Hazard Ratio [HR] = 0.810, 95% CI: 0.517–1.27) or infant HIV exposure (HR = 1.47, 95% CI: 0.859–2.53). Maternal SARS-CoV-2 was associated with a two-fold increased risk of infant infection (HR = 2.31, 95% CI: 1.08–4.94). Few participants (13% mothers, 33% infants) had symptoms; no participant experienced severe COVID-19 or death. Seroreversion occurred in about half of mothers and infants. SARS-CoV-2 sequences obtained from stool were related to contemporaneously circulating variants.

**Conclusions:**

These data indicate that postpartum Kenyan women and their infants were at high risk for SARS-CoV-2 infection and that antibody responses waned over an average of 8–10 months. However, most cases were asymptomatic and healthy women living with HIV did not have a substantially increased risk of infection or severe COVID-19.

## Introduction

To date, SARS-CoV-2 has infected >327,000 people and caused >5,600 deaths in Kenya [1]. Kenya’s first COVID-19 case was reported on March 12, 2020 [2] and generalized community spread was recognized by May 2020, which was followed by several waves of infections with peak rates in July 2020, November 2020, March 2021, August 2021 (delta variant dominant), and January 2022 (omicron variant dominant) [1, 3]. Surveillance suggests most diagnosed SARS-CoV-2 infections in Kenya have been asymptomatic [1]. Like most global regions, Kenya has experienced higher numbers of hospitalizations and deaths during later waves of infections [1, 3], concurrent to emergence of several variants of concern [4].

Kenya has an estimated 1.5 million adults and children living with HIV [5], and there are limited data on the epidemiology of SARS-CoV-2 infection in this population. Several studies and meta-analyses suggest people living with HIV may have an increased risk of COVID-19 mortality, which is higher among those not on ART [6–15]. Persistent inflammation and immune dysregulation are hallmarks of HIV that are only partially resolved by suppressive antiretroviral therapy (ART) [16], and can result in pulmonary, cardiac, and other comorbidities that are risk factors for severe COVID-19 [17]. HIV-induced immune system dysfunction may also directly increase risk of COVID-19 through delayed or inhibited adaptive immune responses or exacerbation of the inflammation [14]. More research is needed—especially from regions with high HIV burden—to understand whether COVID-19 risk remains elevated in individuals with effectively managed HIV infection, whose immune profiles may be more similar to individuals who do not have HIV.

Little is currently known about the risks and outcomes of SARS-CoV-2 infection among postpartum women or HIV-exposed uninfected (HEU) infants. Immunologic changes postpartum may increase susceptibility to SARS-CoV-2 infection or severity of COVID-19. Additionally, HEU infants, which have a nearly two-fold higher risk of overall mortality than HIV-unexposed uninfected infants (HUU) [18–20], may also be at higher risk of severe COVID-19. However, existing data are limited to the effects of SARS-CoV-2 infection during pregnancy or the neonatal period, and there is a lack of comprehensive data among postpartum women living with HIV and HEU infants.

To address these gaps, we assessed the incidence, risk factors, and symptomatology of SARS-CoV-2 infection among postpartum women, both living with HIV and HIV-uninfected, and their infants who were already participating in a longitudinal cohort study in Nairobi, Kenya during the COVID-19 pandemic.

## Methods

Human subjects approvals for all study procedures were obtained from the Kenyatta National Hospital-University of Nairobi Ethics and Research Committee (P472/07/2018) and the University of Washington Institutional Review Board (STUDY00004006). All participants provided an initial written informed consent for participation in the parent cohort study; an additional written informed consent was required for SARS-CoV-2 serology testing. Additional information regarding the ethical, cultural, and scientific considerations specific to inclusivity in global research is included in S1 Checklist.

### Participants and follow-up

This study was nested into the Linda Kizazi Study, a prospective cohort study of the infant virome. Between December 2018-March 2020, 211 pregnant women in their third trimester were recruited from Mathare North Health Centre in Nairobi. Women were eligible if aged 18–40 years, between 28–42 weeks gestation, planning to breastfeed, and, if living with HIV, had received ≥6 months of ART. Exclusion criteria included planned Caesarean section, serious medical condition, and taking antimicrobial or immunosuppressive medication other than for HIV prophylaxis.

All mother-infant pairs were followed from delivery through two years postpartum with clinic visits at week 6, week 10, month 6, and every three months thereafter. At each visit, staff collected information about current and recent symptoms of illness, healthcare visits, diagnoses, medications, and immunizations. A physical exam was performed and samples, including blood and stool, were collected. Data on mothers’ sociodemographic characteristics and health and obstetric history were collected at enrollment. For women living with HIV, CD4 testing was conducted at enrollment and every six months postpartum.

### SARS-Co-V-2 antibody assays

Plasma samples collected between May 1, 2020-February 1, 2022 were tested retrospectively for SARS-CoV-2 antibodies; additionally, pre-pandemic samples were tested to obtain a last-seronegative time point when the first sample tested in the sampling window was seropositive. Samples were tested for detection of total antibodies (IgM/IgA/IgG) to a recombinant SARS-CoV-2 nucleocapsid protein using the Platelia SARS-CoV-2 Total Antibody ELISA (Bio-Rad, Marnes-la-Coquette, France), which had US FDA Emergency Use Authorization at study commencement with reported specificity of 94.9% and a sensitivity of 97.4% [21]. The study laboratory enrolled in an external quality assessment program with the European Society for External Quality Assessment (Heidelberg, Germany) and successfully passed two cycles of proficiency test panels.

The final ELISA result was based on the ratio of the optical density (OD) value of the sample to the mean OD value of the cut-off controls. The result was considered negative for SARS-CoV-2 antibodies if the OD ratio was <0.8, positive if ≥1.0, and equivocal if ≥0.8 and <1.0, according to the manufacturer’s instructions. Samples with equivocal results were retested before final interpretation. Antibody loss was defined by ≥1 negative ELISA result after an initial positive test. For longitudinal modeling of antibody decline, all OD values below the limit of detection (OD ratio = 0.8) were set to 0.4, the midpoint between the lower limit and zero.

### Detection and sequencing of SARS-CoV-2 RNA in stool

Stool samples collected from seropositive participants on or between their last seronegative and first seropositive time points during the first year of the pandemic (January 1-December 31, 2020) were tested for viral RNA. Detailed methods for stool viral RNA extraction, RT-PCR, and sequencing are provided in the S1 Appendix. Briefly, total nucleic acid was extracted from homogenized and filtered stool specimens and quantitative real time PCR (qRT-PCR) was performed using the QuantStudio 3 Real-Time system (Applied Biosystems) [22]. Full-length SARS-CoV-2 genome sequencing was attempted on all qRT-PCR-positive samples. Consensus sequences were called using iVar (version 1.0; parameters -q 20, -t 0.75, -m 20, -n N) [23]. Lineages were assigned using pangolin (version 2.3.8) [24]. Sequence alignments were performed with MAFFT (version 7.471) [25] and phylogenetic reconstruction performed with iqtree with 1000 ultrafast bootstraps [26]. Phylogeny was visualized using FigTree (version 1.4.4) [27]. Sequences used in phylogenetic analysis include 500 randomly selected global sequences from GISAID [28], the Wuhan1 reference genome, and the two genomes sequenced in this study (GISAID accession numbers EPI_ISL_2771497 and EPI_ISL_2771498).

### Statistical analyses

Kaplan-Meier survival analysis was used to estimate the incidence of SARS-CoV-2 infection among mothers and infants separately. Incidence rates (IRs) were calculated as the number of first positive antibody tests per 1000 person-days at risk. Mothers’ time at risk was set to begin on May 1, 2020, when generalized community transmission began in Kenya [1], and infants’ time at risk began either on May 1, 2020 or their date of birth if born later. All participants’ time at risk ended at the estimated time of SARS-CoV-2 infection or the date of their last negative serology test unless otherwise noted. Time of infection was estimated as the midpoint between a participant’s last negative serology test or May 1, 2020 (whichever was later) and their first positive serology test. The log-rank test was used to compare time to infection between women living with HIV and HIV-uninfected women, HEU and HUU infants, and infants whose mother was ever versus never SARS-CoV-2 seropositive. No infants acquired HIV infection.

Cox proportional hazards regression models were used to assess correlates of SARS-CoV-2 infection, both overall and stratified by HIV status/exposure. Growth measures were obtained at infants’ last study visit before May 1, 2020 and include continuous weight-for-age (WAZ), height-for-age (HAZ), and weight-for-height (WHZ) z-scores and the corresponding outcomes of underweight (WAZ <-2), stunting (HAZ <-2), and wasting (WHZ <-2). Z-scores were calculated using the -zanthro- command in Stata using the WHO standard reference and adjusting for sex.

Generalized estimating equations with the log link and an independent correlation structure were used to evaluate the relative risk (RR) of symptoms of COVID-19 associated with participants’ first positive serology result. The outcome was defined as report of ≥1 symptom of COVID-19 experienced either at the time of the visit or since their most recent prior visit (typically a three-month window). Symptoms included those listed by the US Centers for Disease Control and Prevention (CDC), including fever or chills, cough, shortness of breath or difficulty breathing, fatigue, muscle or body aches, headache, new loss of taste or smell, sore throat, congestion or runny nose, nausea or vomiting, and/or diarrhea [29]. Data from both clinic-based and home-based (if applicable) visits were included. All time points prior to the first positive serology test were considered SARS-CoV-2 negative visits for calculation of RRs.

All analyses were conducted in Stata (version 17; StataCorp, College Station, TX, USA) using two-sided tests with a significance level of α = 0.05.

## Results

### Participant characteristics

There were 126 mothers and 125 infants tested for SARS-CoV-2 antibodies. Median age of the mothers was 28 years, most women (90%) were currently married, and fewer than half (41%) were employed. Fewer women living with HIV than HIV-uninfected women were married (86% vs 94%). Although more women living with HIV than HIV-uninfected women were employed (47% vs 36%), their median income was lower (10 vs 27 USD per week). Among women living with HIV, median time on ART on May 1, 2020 was 5 years and median CD4 count at the last attended visit before May 1, 2020 was 565 cells/μl (Table 1).

**Table 1:** Participant characteristics.

		All	HIV-uninfected mothers		Mothers living with HIV	
	N	Median (IQR) or no. (%)	N	Median (IQR) or no. (%)	N	Median (IQR) or no. (%)
**Maternal characteristics**						
Age on May 1, 2020 (years)	126	28 (25, 32)	62	27 (23, 31)	64	30 (27, 33)
Employed	126	52 (41.3)	62	22 (35.5)	64	30 (46.9)
Weekly income, if employed (USD)	39	15 (8, 30)	18	27 (15, 30)	21	10 (8, 20)
Number of people per room in house	125	3 (2, 4)	62	3 (2, 4)	63	3 (2–4)
Currently married	126	113 (89.7)	62	58 (93.6)	64	55 (85.9)
Number of prior live births^a^	126	2 (1, 2)	62	1 (0, 2)	64	2 (1, 3)
CD4 count at last study visit before May 1 (cells/μl)	==	==	==	==	63	565 (457, 740)
Years on ART as of May 1	==	==	==	==	64	5 (3, 8)
**Infant characteristics**						
Sex assigned at birth	125		61		64	
Female		58 (46.4)		30 (49.2)		28 (43.8)
Male		67 (53.6)		31 (50.8)		36 (56.3)
Preterm birth^b^	105	6 (5.7)	55	3 (5.5)	50	3 (6.0)
Low birth weight^c^	104	1 (1.0)	54	1 (1.9)	50	0 (0.0)
Age on May 1 (months)	119^d^	6.3 (3.9, 9.2)	61	6.8 (4.4, 9.3)	58	5.8 (3.2, 8.3)
WAZ at last study visit before May 1	92	-0.3 (-1.0, 0.2)	53	-0.2 (-0.5, 0.3)	39	-0.6 (-1.2, -0.1)
Underweight at last study visit before May 1	92	2 (2.2)	53	0 (0.0)	39	2 (5.1)
HAZ at last study visit before May 1	92	-0.8 (-1.4, -0.1)	53	-0.6 (-1.3, 0.1)	39	-1.1 (-1.6, -0.6)
Had stunting at last study visit before May 1	92	13 (14.1)	53	6 (11.3)	39	7 (18.0)
WHZ at last study visit before May 1	36	-0.2 (-0.9, 0.6)	22	-0.4 (-1.0, 0.6)	14	-0.2 (-0.6, 0.9)
Had wasting at last study visit before May 1	36	2 (5.6)	22	1 (4.6)	14	1 (7.1)
Currently breastfed at last study visit before May 1	95	92 (96.8)	53	52 (98.1)	42	40 (95.2)

IQR = interquartile range; USD = United States dollars; WAZ = weight-for-age z-score; HAZ = length for age z-score; WHZ = weight-for-height z-score.

^a^ Prior to birth of infant enrolled in study.

^b^ Delivery <37 weeks gestation.

^c^ Defined as <2500g.

^d^ Excludes six HEU infants born after May 1, 2020.

Of the 125 infants, 54% were male, 6% were born preterm and 1% had low birth weight. The median age of infants on May 1, 2020 was 6.3 months. At their last study visit prior to May 1, 2020, 14% of infants had stunting, 2% were underweight, and 6% had wasting; a greater proportion of HEU infants than HUU infants had stunting (18% vs 11%, respectively).

### Incidence of SARS-Co-V-2 infection in postpartum women and their infants

**Fig 1:**
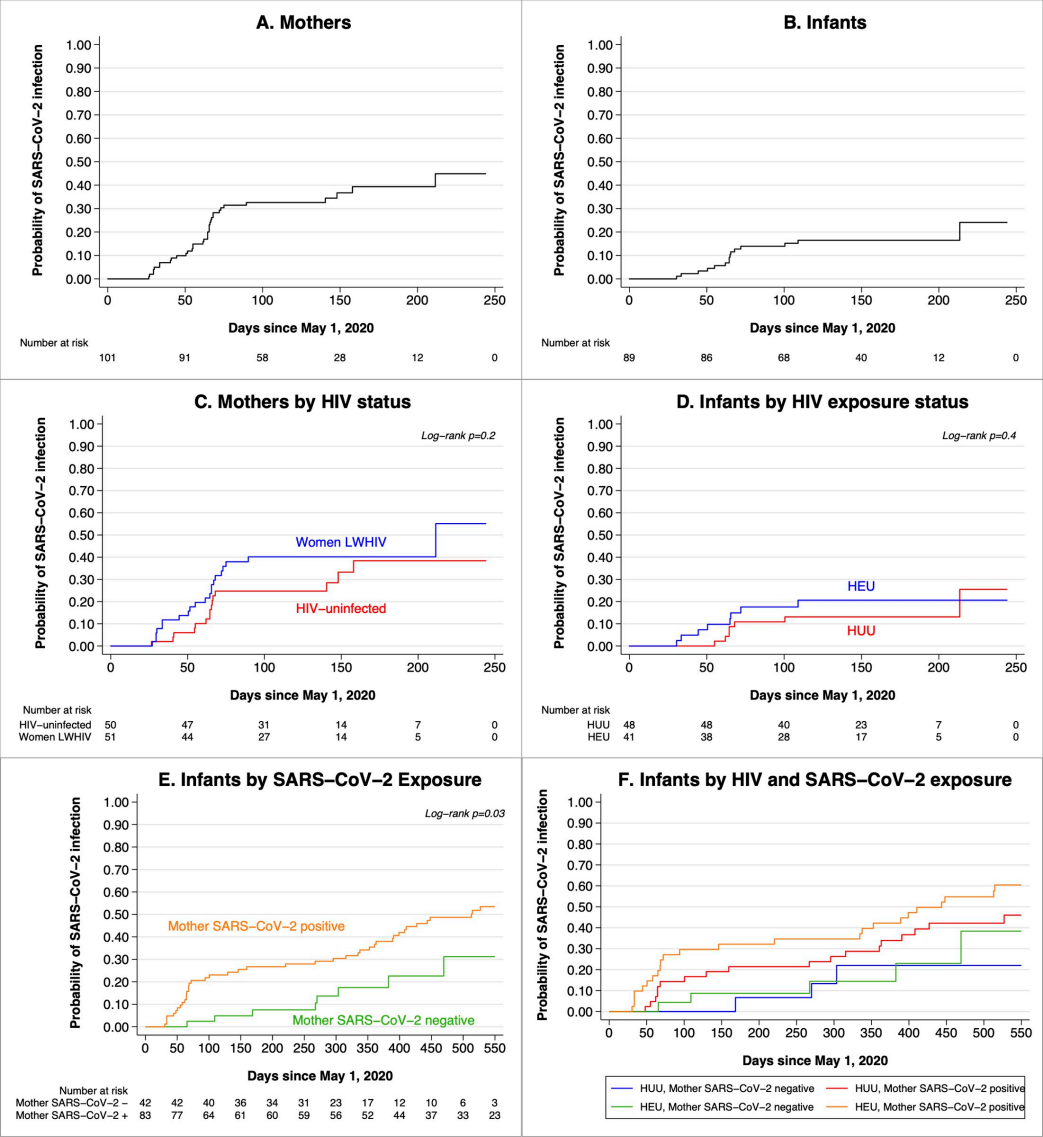
SARS-CoV-2 acquisition from May 1, 2020-February 1, 2022 in postpartum Kenyan women and their infants. Kaplan-Meier hazard functions for participants’ estimated date of infection are shown for (A) all mothers and (B) all infants, (C) mothers stratified by HIV status, (D) infants stratified by HIV exposure, (E) infants stratified by maternal SARS-CoV-2 infection, and (F) infants stratified by maternal HIV and SARS-CoV-2 infection. HEU = HIV-exposed uninfected, HUU = HIV-unexposed uninfected. All women enter the at-risk period on May 1, 2020; infants enter the risk period either at May 1 or on their date of birth, if after May 1. Timing of SARS-CoV-2 infection is estimated as the midpoint between the last negative and the first positive antibody test; for participants whose last negative antibody test was prior to May 1, timing of infection is estimated as the midpoint between May 1 and the first positive test. In (E) and (F), infants’ time at risk is censored on November 1, 2021 since no infants with a SARS-CoV-2 negative mother remained in follow-up for comparison.

Eighty-three cases of SARS-CoV-2 infection were identified among the 126 mothers (66%) and in 55 of the 125 infants (44%; Fig 1). Three of the HIV-uninfected mothers first tested positive before the start of the at-risk period and were excluded from analyses. All other cases first tested positive after May 1, 2020.

The IR among individuals who tested positive after May 1, 2020 was 2.48 (95% CI: 1.99–3.09) per 1000 person-days among mothers and 1.24 (95% CI: 0.951–1.61) among infants (Table 2). The incidence of SARS-CoV-2 infection did not differ significantly between women living with HIV and HIV-uninfected women (HR = 0.810, 95% CI: 0.517–1.27; p = 0.4) or HEU versus HUU infants (HR = 1.47, 95% CI: 0.859–2.53; p = 0.2). Infants whose mothers were SARS-CoV-2 seropositive were just over two times more likely to acquire infection (Hazard Ratio [HR] = 2.30, p = 0.032).

**Table 2:** Incidence of SARS-CoV-2 infection in postpartum Kenyan women and their infants from May 1, 2020 to February 1, 2022.

	Cases	Person-days^1^	Cases per 1000 person-days (95% CI)
**All mothers (N = 123** ^**2**^ **)**	**80**	**32,275**	**2.48 (1.99, 3.09)**
HIV-uninfected (n = 59)	39	14,618	2.67 (1.95, 3.65)
Living with HIV (n = 64)	41	17,658	2.32 (1.71, 3.15)
**All infants (N = 125)**	**55**	**44,398**	**1.24 (0.951, 1.61)**
***By HIV exposure***			
HIV-unexposed (n = 61)	22	22,318	0.986 (0.649, 1.50)
HIV-exposed (n = 64)	33	22,081	1.49 (1.06, 2.10)
***By SARS-CoV-2 exposure*** ^3^	**50**	**43,493**	**1.15 (0.871–1.52)**
Mother SARS-CoV-2 antibody-negative (n = 42)	8	13,698	0.584 (0.292, 1.17)
Mother SARS-CoV-2 antibody-positive (n = 83)	42	29,795	1.41 (1.04, 1.91)

CI = confidence interval. HR = hazard ratio.

^1^ Person time in days from May 1, 2020 or date of birth for infants born after May 1, 2020.

^2^ Excludes three HIV-uninfected mothers who tested positive before May 1, 2020.

^3^ Infants’ time at risk is censored on November 1, 2021 since no infants with a SARS-CoV-2 negative mother remained in follow-up for comparison.

There was one SARS-CoV-2 seropositive infant for whom a false-positive test due to maternal antibody transfer during pregnancy could not be ruled out. The infant was born to a mother living with HIV who tested negative for SARS-CoV-2 antibodies at enrollment at 28 weeks gestation but was seropositive at her first follow-up visit at 6 weeks postpartum. The infant’s first available blood sample, collected at week 10, was SARS-CoV-2 positive (OD ratio = 1.66); subsequent samples were positive at month 6 (OD ratio = 1.26) and negative at month 9 (OD ratio = 0.371), month 12 (OD ratio = 0.237), and month 18 (OD ratio = 0.0616). Exclusion of this infant from analyses did not substantially change incidence estimates (overall: 1.22, 95% CI: 0.932–1.59 per 1000 person-days; HEU: 1.45, 95% CI: 1.03–2.05 per 1000 person-days) or the hazard of infection associated with HIV (HR = 1.43, 95% CI: 0.831–2.46) or maternal SARS-CoV-2 infection (HR = 2.01, 95% CI: 0.975–3.16).

### Duration of SARS-CoV-2 antibody detection

**Fig 2:**
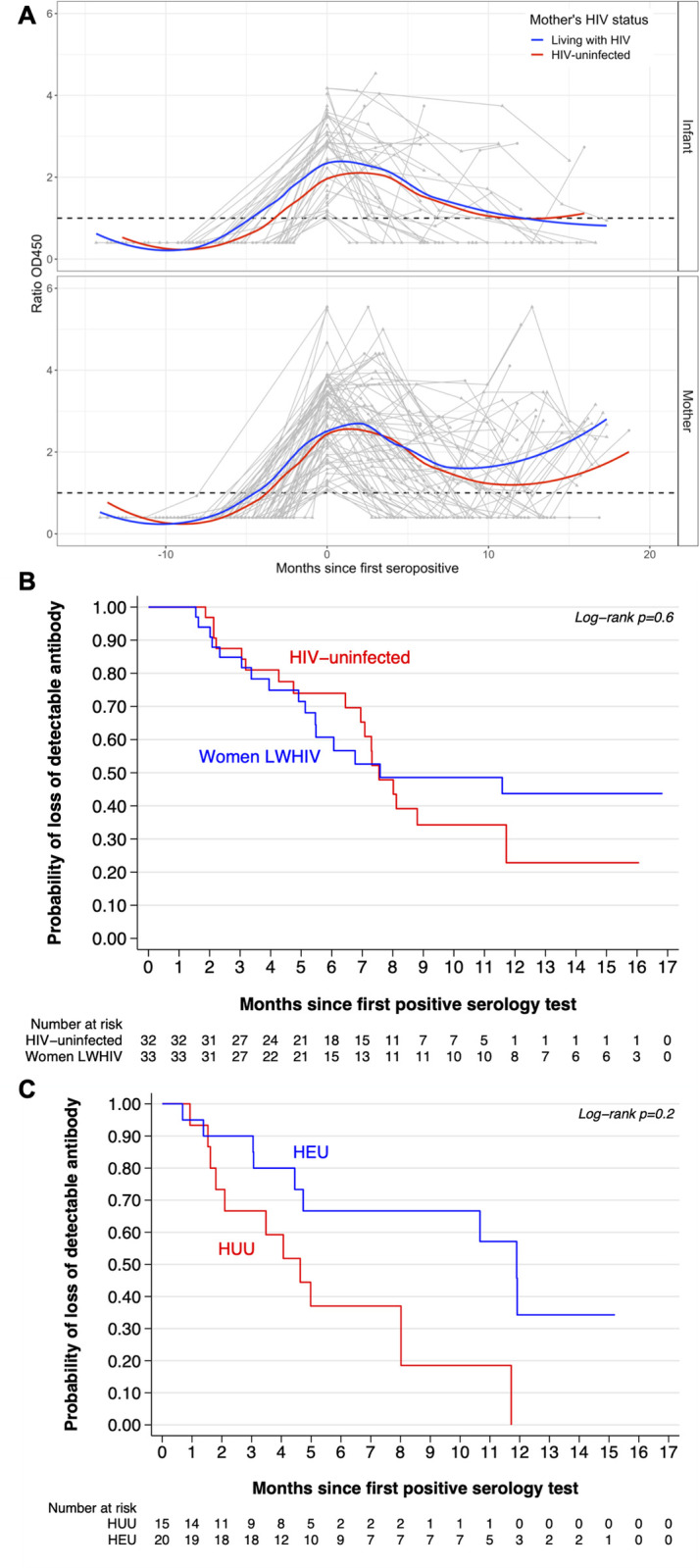
Detection of SARS-CoV-2 antibody among mothers and infants over time. (A) SARS-CoV-2 antibody levels over time relative to the first seropositive time point (0 months). Individual patterns in infants (top) and mothers (bottom) are shown in grey. Grouped by maternal HIV status, running means are shown for HIV-uninfected women or HIV-unexposed infants in black and women living with HIV or HIV-exposed infants in red. Limit of detection denoted by dashed vertical line. (B) and (C) are Kaplan-Meier hazard functions for participants’ estimated time to loss of detectable antibodies stratified by maternal HIV status and infant HIV exposure, respectively. HEU = HIV-exposed uninfected, HUU = HIV-unexposed uninfected. The risk period for loss of detectable antibody begins at the participant’s first positive serology test and ends either at the time of loss of detectable antibodies (estimated as the midpoint between the last positive test and first negative test after a positive test) or at the time of the most recent positive test.

SARS-CoV-2 antibody changes over time were examined in all participants with ≥1 test following their first positive (65 mothers and 35 infants). Antibody levels declined rapidly in both mothers and infants, regardless of HIV exposure, waning below the limit of detection in 34 (52%) women and in 20 (57%) infants (Fig 2A). The mean time between participants’ first positive serology test and loss of antibody detection was 9.7 months (95% CI: 8.1–11.2) for mothers and 8.1 (95% CI: 6.2–10.0) months for infants. Time to an antibody-negative test was not significantly different between women living with HIV (8.8 months, 95% CI: 6.8–10.7) and HIV-uninfected women (10.1 months, 95% CI: 7.8–12.4; long-rank p = 0.6) but was significantly shorter among HUU infants (5.5 months, 95% CI: 3.4–7.5) compared to HEU infants (10.0 months, 95% CI: 7.5–12.5; log rank p = 0.022; Fig 2B and 2C).

Among 18 mother-infant pairs where both mother and infant were first SARS-CoV-2 seropositive at the same visit and had ≥1 follow-up sample, 5 pairs remained concordant positive in all follow-up samples, 3 mothers lost antibody while their infant remained positive, 4 infants lost antibody while their mother remained positive, and in 6 cases both mother and infant lost antibody, either concurrently (2 cases) or at different time points (4 cases; S1 Fig). Five mothers and 2 infants tested positive again after showing antibody loss from their initial infection.

### SARS-CoV-2 viral RNA isolation from stool

**Fig 3:**
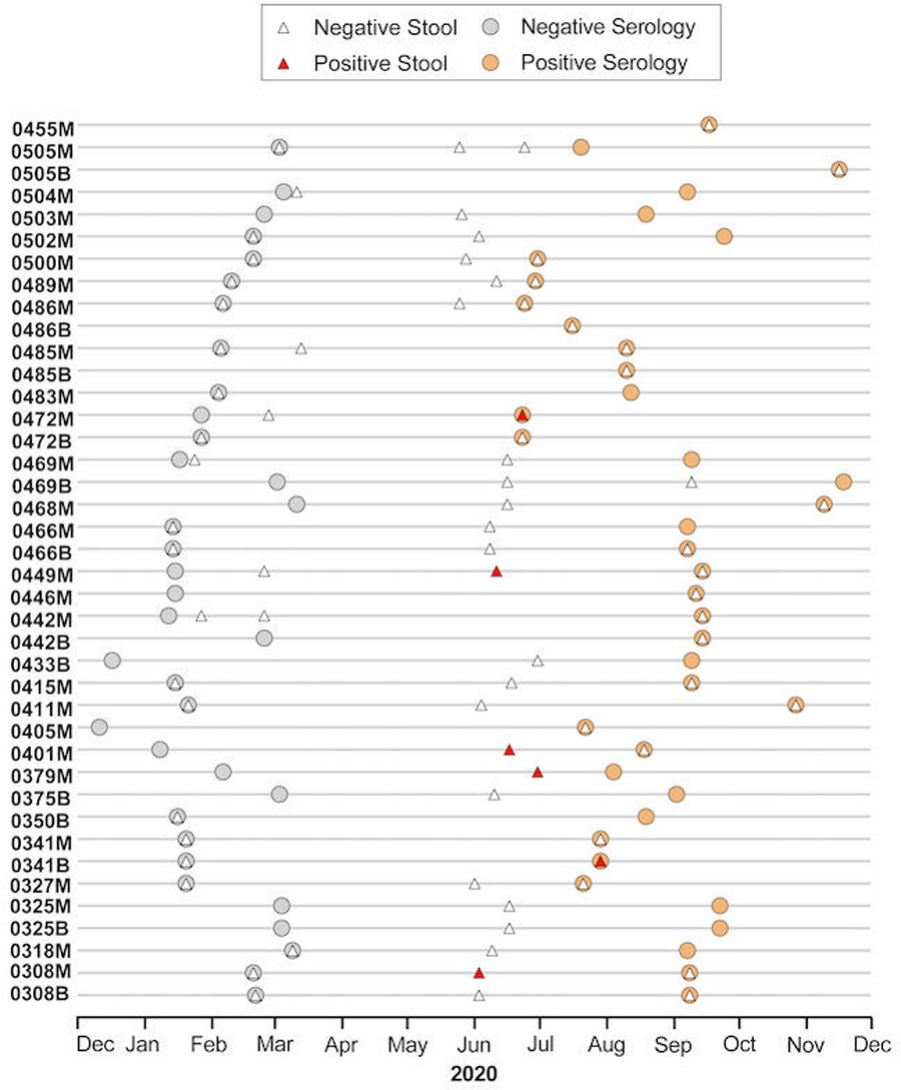
SARS-CoV-2 serology and stool viral RNA results over calendar time. Results of SARS-CoV-2 serology and quantitative real-time PCR testing of stool samples from Linda Kizazi participants that first tested seropositive and had ≥1 available stool sample collected between May 1-December 31, 2020. Anonymized ID numbers on y-axis for mothers (M) and infants (B). Grey circles indicate date of last seronegative serology test and orange circles indicate date of first seropositive sample. White triangles represent SARS-CoV-2 RNA-negative and red triangles represent RNA-positive stool samples. Calendar time is on the x-axis.

Twenty-seven mothers and 13 infants had stool samples available between their last seronegative and first seropositive time points for SARS-CoV-2 RNA testing. Of these, 5 mothers (19%) and one infant (8%) had detectable SARS-CoV-2 RNA (Fig 3). Two participants’ RNA-positive stool sample was collected on the same day as their first SARS-CoV-2 seropositive blood sample; the other 4 were RNA-positive prior to the first positive serology test. Median stool SARS-CoV-2 RNA level was 60,989 copies/ml (IQR 27,953–1,646,080) in mothers; the one infant’s RNA level was 10,181 copies/ml. SARS-CoV-2 genome sequencing was performed on six RNA-positive stool samples. High-quality whole genome sequences were obtained from two samples (S2 Fig). Both sequences were classified as B.1 lineage, a predominant global lineage that was also identified in Kenya in 2020.

### Correlates of SARS-CoV-2 infection

Older age was associated with increased risk of SARS-CoV-2 infection among women living with HIV (HR = 1.13, 95% CI: 1.05–1.22) but not among HIV-uninfected women (HR = 1.03, 95% CI: 0.974–1.09; Table 3). The risk of SARS-CoV-2 infection among all women also increased 22% with each additional person per room in the home (HR = 1.22, 95% CI: 1.04–1.42) and 19% with each prior live birth (HR = 1.19, 95% CI: 1.02–1.40), though these associations appeared to be influenced primarily by increased risk among women living with HIV. No other maternal characteristics were significantly associated with SARS-CoV-2 infection. Overall, infants whose mother was ever SARS-CoV-2 seropositive were two times more likely to have SARS-CoV-2 infection themselves (HR = 2.07, 95% CI: 1.00–4.27). No other infant characteristics were significantly associated with SARS-CoV-2 infection.

**Table 3:** Correlates of SARS-CoV-2 infection in mothers and infants.

	All			HIV-uninfected mothers			Mothers living with HIV		
Cofactor	N	HR (95% CI)	p-value	N	HR (95% CI)	p-value	N	HR (95% CI)	p-value
**Mothers (N = 123** ^**a**^ **)**									
Age on May 1 (years)	123	**1.06 (1.02, 1.11)**	**0.006**	59	1.03 (0.974, 1.09)	0.3	64	**1.13 (1.05, 1.22)**	**0.001**
Employed	123	0.801 (0.509, 1.26)	0.3	59	0.962 (0.501, 1.85)	0.9	64	0.673 (0.353, 1.28)	0.2
Weekly income, if employed (USD)	38	1.00 (0.972, 1.03)	>0.9	17	0.996 (0.957, 1.04)	0.9	21	0.990 (0.930, 1.05)	0.8
Number of people per room in house	**122**	**1.22 (1.04, 1.42)**	**0.013**	59	1.16 (0.926, 1.45)	0.2	**63**	**1.27 (1.02, 1.59)**	**0.03**
Currently married	123	1.58 (0.686, 3.66)	0.3	59	2.78 (0.379–20.4)	0.3	64	1.22 (0.472, 3.14)	0.7
Number of prior live births^b^	**123**	**1.19 (1.02, 1.40)**	**0.03**	59	1.17 (0.950, 1.44)	0.1	64	1.26 (0.984, 1.62)	0.067
Living with HIV	123	0.810 (0.517, 1.27)	0.4	--	--	--	--	--	--
Last CD4 count before May 1 (cells/μl)^c^	--	^--^	--	--	--	--	63	1.01 (0.993–1.02)	0.4
Years on ART as of May 1	--	--	--	--	--	--	64	1.07 (0.973, 1.19)	0.2
**Infants (N = 125)**									
Female sex assigned at birth	125	0.931 (0.547, 1.58)	0.8	61	1.03 (0.444, 2.37)	>0.9	64	0.950 (0.474, 1.91)	0.9
HIV exposed	125	1.47 (0.859, 2.53)	0.2	--	--	--	--	--	--
Mother ever SARS-CoV-2 seropositive^d^	125	**2.31 (1.08, 4.94)**	**0.03**	61	2.18 (0.637, 7.46)	0.2	64	2.50 (0.945, 6.60)	0.07
Age on May 1 (months)	119^e^	1.03 (0.951, 1.13)	0.4	61	1.03 (0.903–1.18)	0.6	58	1.05 (0.947, 1.17)	0.3
WAZ at last visit before May 1	92	0.947 (0.691, 1.30)	0.7	53	0.938 (0.590, 1.49)	0.8	39	1.08 (0.664, 1.76)	0.8
HAZ at last visit before May 1	92	0.937 (0.711, 1.23)	0.6	53	0.838 (0.579, 1.21)	0.3	39	1.27 (0.726, 2.23)	0.4
WHZ at last visit before May 1	36	0.995 (0.654, 1.51)	>0.9	22	1.07 (0.617, 1.84)	0.8	14	0.919 (0.497, 1.70)	0.8
Currently breastfed at last visit before May 1	95	0.632 (0.0848, 4.71)	0.7	53	--	--	42	0.389 (0.0484, 3.13)	0.4

HR = Hazard ratio; CI = Confidence interval; WAZ = weight-for-age z-score; HAZ = length for age z-score; WHZ = weight-for-height z-score.

^a^ Excludes three HIV-uninfected mothers who tested positive before May 1, 2020.

^b^ Prior to birth of infant enrolled in study.

^c^ HR is difference for every 10 cells/μl.

^d^ Infants’ time at risk is censored on November 1, 2021 since no infants with a SARS-CoV-2 negative mother remained in follow-up for comparison

^e^ Excludes six HEU infants born after May 1, 2020.

### Symptoms of SARS-CoV-2 infection

There were no hospitalizations or deaths in the study cohort due to COVID-19. Of 78 SARS-CoV-2 antibody-positive mothers with symptom data available at the time of their first seropositive visit or since their most recent visit prior, 10 (13%) reported ≥1 symptom of COVID-19. Of 54 SARS-CoV-2 antibody-positive infants with symptom data, ≥1 symptom of COVID-19 was reported for 18 (33%). In mothers, symptoms were about two times more likely at the first SARS-CoV-2 seropositive visit (RR = 2.29, 95% CI: 1.22–4.27; p = 0.010) and in infants, symptoms were nearly three times more likely at the first SARS-CoV-2 seropositive visit (RR = 2.75, 95% CI: 1.81–4.17; p<0.001) compared to earlier seronegative visits.

## Discussion

In this cohort of postpartum Kenyan women and their infants, 66% of mothers and 44% of infants experienced SARS-CoV-2 infection between May 1, 2020-February 1, 2022, most of which was asymptomatic (87% and 67%, respectively). There was no significant association between maternal HIV status and either maternal or infant SARS-CoV-2 infection risk. Together, our data suggest that the initial two waves of COVID-19 in Kenya likely resulted in high rates of asymptomatic infection in postpartum women and their infants.

This study is one of few that have examined the incidence of SARS-CoV-2 among postpartum women and their infants, including those living with HIV. Our findings among these women in living in a densely populated, urban neighborhood of Nairobi, are consistent with seroprevalence from a general population-based cross-sectional survey of households in November 2020, which found an age- and sex-adjusted seroprevalence of 34.7% in Nairobi County overall and 52.7% in the Mathare sub-county where the Linda Kizazi Study cohort resides [30]. Our slightly higher estimates are likely due to the delta and omicron waves that occurred subsequent to the above described 2020 survey. Like our study, the survey also noted a higher seroprevalence among adults (ages 20–59; 38.6%) than children (ages 0–9; 19.5%).

Consistent with the rapid increase of COVID-19 in Kenya after the first wave of infections, our study with data from 2020–2022 we found a greater number of SARS-CoV-2 infections than a study of adult blood donors conducted only early in the pandemic between April-June 2020, which showed just 7.8% of Nairobi County residents were seropositive [31]. Our study also found higher seroprevalence than a study of adults both living and not living with HIV from Western Kenya; retrospective samples from January-March 2020 showed that only 3.3% of participants had SARS-CoV-2 antibodies, though like our data there was no significant difference in infection between individuals with and without HIV (3.1% vs 4.0%, respectively) [32]. Additionally, our cohort’s incidence of SARS-CoV-2 infection was substantially higher than that of mother-infant pairs presenting with symptoms of COVID-19-like illness in a contemporaneous Siaya County, Kenya-based cohort study, which measured an incidence of 1.8 cases per 1000 person-months among postpartum women and 0.9 per 1000 person-months among infants [33]. This difference may be due to the high proportion of asymptomatic infections in Kenya.

Though fewer infants than mothers in our study experienced SARS-CoV-2 infection, infants whose mothers acquired SARS-CoV-2 infection were almost three times as likely to become infected compared to infants not exposed to maternal SARS-CoV-2 infection. A recent meta-analysis of 54 intra-household transmission studies including >70,000 participants estimated a secondary attack rate of 17% overall, with rates higher in adults compared to children [34]. Though our analysis was unable to determine the household index case for participants in our study, the very high rate of infant infections and association with maternal SARS-CoV-2 infection suggest infant infections were likely acquired from their mother or a shared index case.

In this cohort, we did not find a significantly different risk of SARS-CoV-2 infection due to maternal HIV infection and CD4 count was not associated with risk of infection among women living with HIV. However, the differential risk of SARS-CoV-2 infection was 13% greater with each additional year of age among women living with HIV. This finding is consistent with existing evidence that older age is associated with increased risk of SARS-CoV-2 infection and COVID-19 morbidity and mortality both in Kenya and globally [35–39].

Despite the low overall prevalence of symptoms among seropositive participants, the relative risk of symptoms was two times higher among mothers and almost three times higher among infants at the first seropositive visit compared to earlier visits. There were no cases of severe COVID-19 or death among women in our cohort, which could be due in part to the young age of the participants. A study of the COVID-19 pandemic’s effects on children in sub-Saharan Africa (SSA) postulated that the larger populations of people <20 years in the region (52.7%) compared to Asia (31.2%), North America (24.5%), and Europe (21.2%) could partially explain the relatively low COVID-19 case burden and case fatality rate in this region [40]. However, it is also possible that limited surveillance and reporting in SSA underestimates the true burden of COVID-19 morbidity and mortality. Furthermore, the Linda Kizazi Study’s strict eligibility criteria generated a cohort of women that were mostly young, in good health, well-engaged in medical care, and, if living with HIV, had well-controlled HIV infection. All women in the analysis also delivered a healthy infant, another marker of maternal health. Thus, in this cohort of healthy postpartum women, we did not observe a significant effect of HIV co-infection on SARS-CoV-2 infection and few symptoms of COVID-19 overall, as compared to other populations living with HIV, in whom HIV has been associated with increased COVID-19 severity and death [8–14].

In seropositive mothers and infants with additional samples available post-SARS-CoV-2 infection, antibody levels waned over time and just over half (52% of mothers and 57% of infants) had undetectable levels in a mean of 8–10 months. While there was no significant difference in time to loss of detectable antibody due to maternal HIV status, HUU infants experienced a shorter mean time to undetectable antibody than HEU infants. Our data are consistent with reports demonstrating antibody detectability for up to 8–12 months characterized by an initial peak within the first few months followed by a gradual decline [41, 42]. It is possible that mild and asymptomatic infections may have shorter-lived antibody detection than more severe infections [43–45]. In the subset of participants who lost detectable antibody, we noted there were some individuals who tested positive again after loss of detection. It is possible these participants experienced reinfection, but continued follow-up is needed to confirm and characterize reinfection in this cohort and to provide further insights into factors that influence duration of antibody detection.

Our study has several strengths, including prospective longitudinal testing of postpartum women living with HIV and HIV-uninfected women and their infants, and systematic and detailed assessment of clinical symptoms blind to COVID-19 infection status. We did not have viral RNA testing in nasal swabs to confirm serology results or detect acute SARS-CoV-2 infection, although we were able to verify the presence of virus in stool for some cases. Our study also has some important limitations to note, including a modest sample size, which may have precluded the detection of weaker associations. As discussed above, our selected population is not representative of the more heterogenous population of Kenyan postpartum women, women living with HIV, and infants due to our strict eligibility criteria. Many infants and mothers first tested SARS-CoV-2 antibody positive at the same visit, making it difficult to determine direction of infection, or to know whether both may have acquired infection from a shared index case. Three-month sampling intervals and missed visits during the start of the pandemic, while study procedures were being revised to be “no-contact” in accordance with Kenya Ministry of Health guidance, preclude precise ascertainment of timing of infection or detection of virus in some cases.

In summary, our data demonstrate high rates of asymptomatic and mildly symptomatic COVID-19 among healthy postpartum women with or without HIV co-infection between May 2020 and February 2022 in Kenya. Waning antibodies raise the possibility that despite high rates of infection, a large proportion of individuals may be susceptible to reinfection [46, 47]. Women living with HIV and their infants were not found to be at a substantially increased risk of COVID-19 compared to HIV-uninfected women and their infants in this cohort, in contrast to other populations living with HIV. Continued practice of preventative measures such as social distancing and masking will remain important until COVID-19 vaccine coverage increases in Kenya.

## Supporting information

S1 Appendix Detailed methods for stool viral RNA extraction, RT-PCR, and sequencing. (DOCX)

S1 Fig SARS-CoV-2 antibody levels over time in mother-infant Pairs. OD ratios show SARS-CoV-2 antibody levels over time in pairs where both mother and infant were first SARS-CoV-2 positive at the same visit and had ≥1 sample available after initial antibody detection. Increased levels of antibody denoted by darker purple shading as shown in key. Positive antibody levels denoted by filled circle, equivocal levels by X, and levels below the limit of detection by empty circles. Mother-infant pairs in which the mother was living with HIV are shown on top with bold red IDs; HIV-uninfected and -unexposed pairs are shown below with black IDs.
(TIF)

S2 Fig SARS-CoV-2 genomes sequenced from Kenyan stool samples. Complete SARS-CoV-2 genomes were sequenced from six RNA-positive stool samples. (A) Phylogenetic analyses of 500 randomly selected SARS-CoV-2 global sequences, the Wuhan1 reference, and the two Kenyan stool-derived genomes (indicated in red) are shown. Clade labels are shown. (B) Next-generation sequencing data statistics of the six Kenyan stool samples.
(TIF)

S1 Checklist Inclusivity in global research. (DOCX)
