# Supplementary figures and images for: HIV and SARS-CoV-2 infection in postpartum Kenyan women and their infants
Source: PLoS One. 2023 Jan 17;18(1):e0278675. doi: 10.1371/journal.pone.0278675 (PMC9844875; doi:10.1371/journal.pone.0278675)

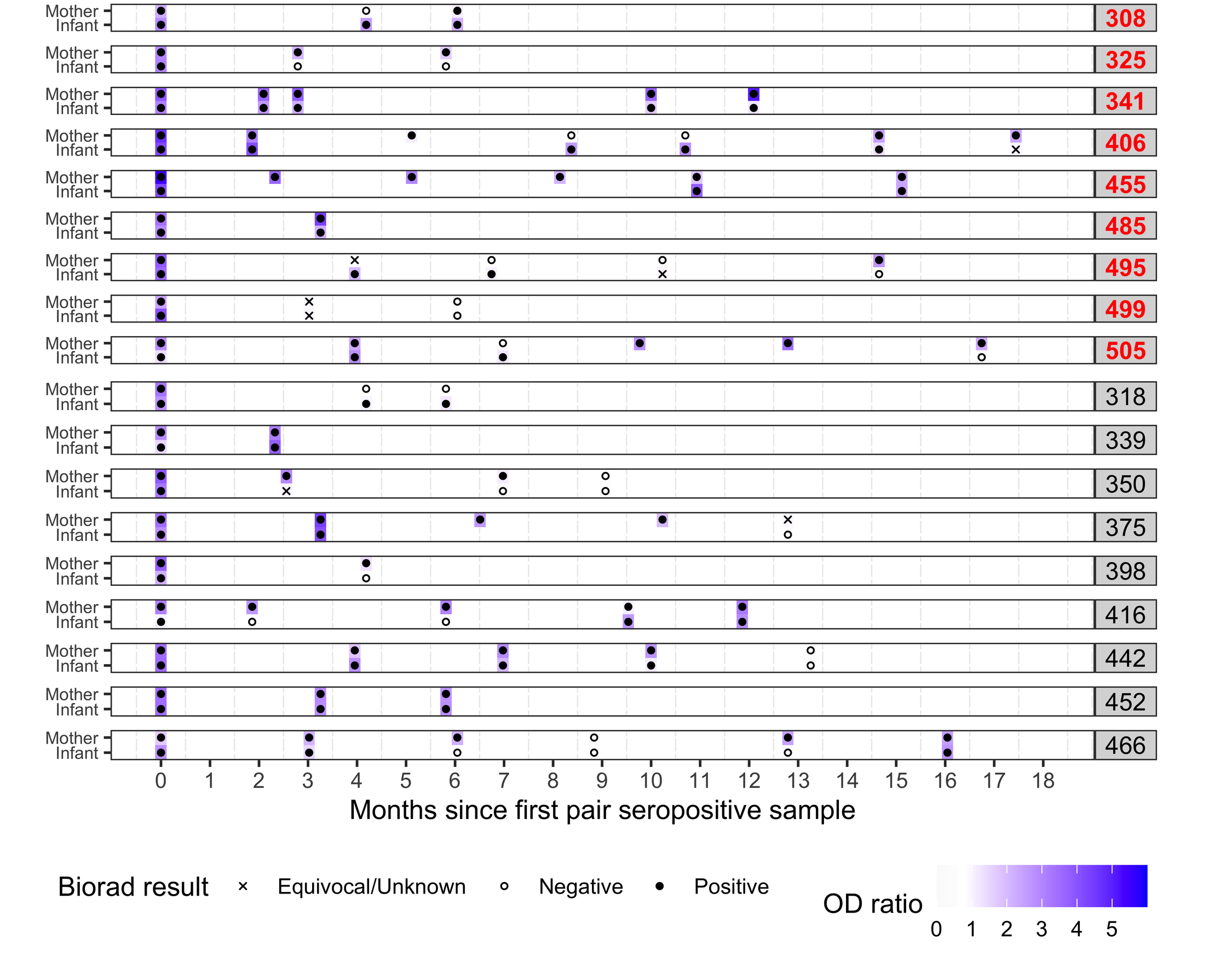

Supplement: S1 Fig — OD ratios show SARS-CoV-2 antibody levels over time in pairs where both mother and infant were first SARS-CoV-2 positive at the same visit and had ≥1 sample available after initial antibody detection. Increased levels of antibody denoted by darker purple shading as shown in key. Positive antibody levels denoted by filled circle, equivocal levels by X, and levels below the limit of detection by empty circles. Mother-infant pairs in which the mother was living with HIV are shown on top with bold red IDs; HIV-uninfected and -unexposed pairs are shown below with black IDs. (TIF) [file pone.0278675.s002.tif]

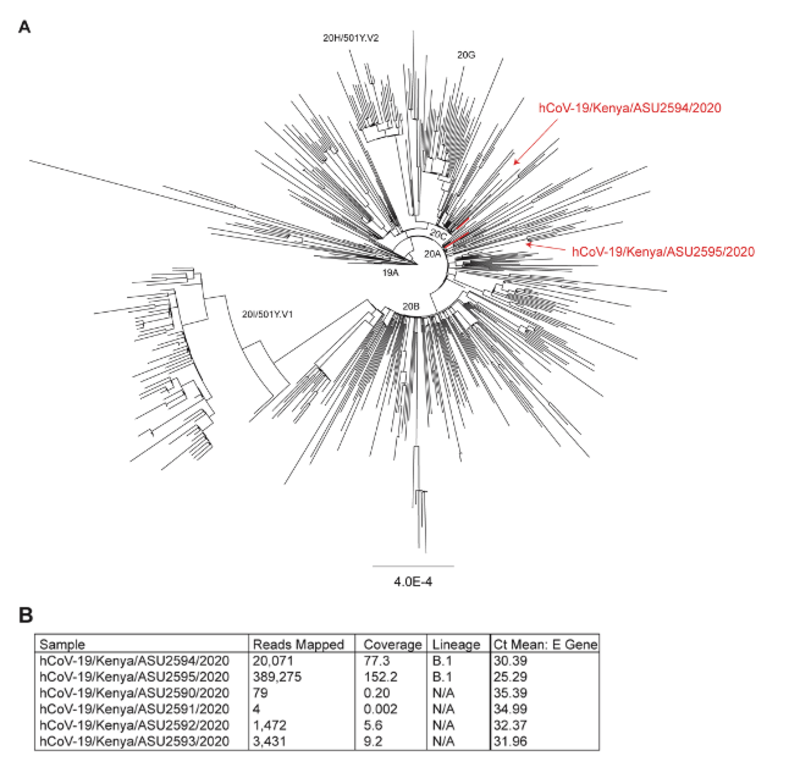

Supplement: S2 Fig — Complete SARS-CoV-2 genomes were sequenced from six RNA-positive stool samples. (A) Phylogenetic analyses of 500 randomly selected SARS-CoV-2 global sequences, the Wuhan1 reference, and the two Kenyan stool-derived genomes (indicated in red) are shown. Clade labels are shown. (B) Next-generation sequencing data statistics of the six Kenyan stool samples. (TIF) [file pone.0278675.s003.tif]
