# Supplementary material for: HIV and SARS-CoV-2 infection in postpartum Kenyan women and their infants
Source: PLoS One. 2023 Jan 17;18(1):e0278675. doi: 10.1371/journal.pone.0278675 (PMC9844875; doi:10.1371/journal.pone.0278675)
Supplement: S1 Checklist — (DOCX) [file pone.0278675.s004.docx]

Inclusivity in global research

PLOS’ policy on inclusivity in global research aims to improve transparency in the reporting of research performed outside of researchers’ own country or community and ensures that PLOS publications reporting global research adhere to high standards for research ethics and authorship. Authors of relevant research articles may be asked to complete the questionnaire below, which outlines ethical, cultural, and scientific considerations specific to inclusivity in global research. This questionnaire may be requested when researchers have travelled to a different country to conduct research, if research uses samples collected in another country, research with Indigenous populations or their lands, or if research is on cultural artefacts. Researchers travelling to another country solely to use laboratory equipment will not normally be required to complete the questionnaire. However, the questionnaire can be requested at the journal’s discretion for any submission – if you have been requested to complete this questionnaire by the PLOS journal you submitted to, please do so.

Please complete the questionnaire below and include this as a Supporting Information file with your manuscript. Note that if your paper is accepted for publication, this checklist will be published with your article in the supporting information files. Please ensure that you reference the checklist in the main body of your manuscript. We suggest adding a subsection ‘Inclusivity in global research’ to your Methods section and adding the following sentence: “Additional information regarding the ethical, cultural, and scientific considerations specific to inclusivity in global research is included in the Supporting Information (SX Checklist)”

The questions have been designed to be applicable to a wide range of study types, and there are subsections for both human subjects research and non-human subjects research. If any of the questions are not relevant to your research please mark them as “N/A” as appropriate.

**Ethical considerations, permits and authorship**

*This section is applicable to all research types.*

Provide details as to who granted permissions and/or consent for the study to take place in the Methods section of your manuscript. This should include the names of **all** ethics boards, governmental organizations, community leaders or other bodies that provided approval for the study. If individuals provided approval refer to these people by their role or title but do not list their name(s).

Reported on page number: 6

If there were any deviations from the study protocol after approval was obtained please provide details of these changes in the Methods section of your manuscript.
Did this study involve local collaborators that are residents of the country where the research was conducted or members of the community studied? If you do not have any authors from said communities, please provide an explanation for this below.

Reported on page number: Not applicable. There were no deviations from the study protocol after approval was obtained.

Yes, the following authors are residents of Kenya, where this research was conducted: Ednah Ojee (co-first author), Judith Adhiambo, Prestone Owiti, Vingent Ogweno, John Kinuthia (co-senior author), and Dalton Wamalwa (co-senior author). Additionally, these individuals have experience working with the study facility and the local community from which study participants were recruited.

Everyone listed as an author should meet PLOS’ criteria for authorship and all individuals who meet these criteria should be included in the author byline, rather than the acknowledgements. Authorship criteria is based on the International Committee of Medical Journal Editors (ICMJE) Uniform Requirements for Manuscripts Submitted to Biomedical Journals - for further information please see here: <https://journals.plos.org/plosone/s/authorship>.

**Human subjects research (e.g. health research, medical research, cross-cultural psychology)**

Did you obtain written informed consent from a representative of the local community or region before the research took place? How did you establish who speaks for the community? Details of written informed consent obtained from study participants should be reported separately in the Methods section of your manuscript.

Prior to study start of the parent cohort, the Linda Kizazi Study, written permission to conduct the study was obtained from the Nairobi City County Director of Health Services. Written permission was also obtained from Mathare North Health Centre, where enrollment and study clinic visits took place.

Meetings with the facility and a community advisory board (CAB) were conducted prior to study start and quarterly thereafter to discuss study procedures, implementation, and results dissemination. The CAB consisted of Mathare North Health Centre facility nurses and administrators, local health administrators, community health workers, HIV peer counselors, and leaders of community groups. If needed, changes to the study were made in response to CAB feedback.

Participants were also provided with contact information for the study staff, study leadership, and the ethical committees overseeing the study (University of Washington Institutional Review Board and Kenyatta National Hospital-University of Nairobi Ethics and Research Committee) so that they may ask information about the study or report any concerns about or consequences of their participation in the study.

How did members of the local community provide input on the aims of the research investigation, its methodology, and its anticipated outcome(s)?

The study aims and methodology were reviewed by and edited in response to feedback from the Kenyatta National Hospital-University of Nairobi Ethics and Research Committee, which ensures research studies are appropriate for individuals in the Nairobi, Kenya area. Additionally, the study leadership team requested feedback on the aims, methodology—including data to be collected and measurement methods—and outcomes from the study field staff, the study facility, and the community advisory board (CAB) prior to beginning the study. Questions, concerns, and suggestions from these key stakeholders were incorporated into the study as modifications to the protocol, which were reviewed and approved by the ethics and research committee and standard operating procedures. Feedback from study participants, field staff, study facility staff, and CAB members has also been incorporated into plans for secondary analyses of study data, which will be disseminated to these key stakeholders upon publication.

When engaging with the local community, how did you ensure that the informed consent documents and other materials could be understood by local stakeholders?

Consent documents were included in the materials shared with the study facility and community advisory board. Prior to study start, the consent forms were developed with Kenya-based study team members and all field staff members provided feedback on the forms before they were finalized for use with participants. Residents of the study catchment area (the Mathare North neighborhood of Nairobi, Kenya) primarily speak English or Kiswahili. We did not enroll individuals who could not speak either English or Kiswahili. All consent materialswere written in plain, layperson language and translated into Kiswahili by a professional translator. Translations were verified by field staff, all of whom were residents of Kenya spoke and read English and Kiswahili. Participants chose which language they preferred for written informed consent procedures. Participants who could not read were consented in the presence of an independent witness who did not conduct the informed consent procedures. Participants were offered a copy of all signed consent documents to take home with them.

Will the findings of the research be made available in an understandable format to stakeholders in the community where the study was conducted (e.g. via a presentation, summary report, copies of publications, etc.)? Please provide details of how this will be achieved.

Yes. Upon publication, the results of this nested analysis will be presented in person to the study facility, the community advisory board, and laboratory partners, medical trainees, and faculty at the University of Nairobi and Kenyatta National Hospital. Copies of the published manuscript will be available to all of these stakeholders. A summary of key results, in lay language, will be made available to Linda Kizazi Study field staff to share with participants at follow-up visits.

**Non-human subjects research using specimens/ animals collected as part of the study, or those housed in archival collections. Examples include archaeology, paleontology, botany and zoology.**

Did the permission you obtained from a local authority to perform the study include an agreement on access to outputs and benefit sharing? This may include procedures to enable fair distribution of the benefits and resources arising from the research performed. Please include any details of Prior Informed Consent and Benefit Sharing Agreements obtained. These may be required by field-specific regulations, for example the Convention on Biological Diversity (CBD) and the associated Nagoya Protocol.

Not applicable.

If the material used in your study was imported, please A) provide the year it was imported and B) indicate whether permits were obtained to import/export the materials used, C) provide details of any permits obtained. If this information is not available, please indicate this.

Not applicable.

If you used archival specimens, please state how the material used in your study was acquired by the institute it is held in and provide details of any permits obtained for the original excavations/ sample collection. If this information is not available, please indicate this.

Not applicable.

How was the potential cultural significance of the materials collected in your study to local communities considered in your research design? Were Indigenous peoples and/or local researchers and institutions involved with archaeological excavations / collection of specimens? If so, please provide a description of their involvement.

Not applicable.

If your manuscript includes photographs of human remains please indicate whether authors obtained permission from descendants or affiliated cultural communities to do so.

Not applicable.
